# Harnessing liquid biopsy to unveil RAS-MEK pathway somatic pathogenic variants in extracranial arterio-venous malformations

**DOI:** 10.1038/s43856-025-01174-1

**Published:** 2025-12-05

**Authors:** Kartik D. Bhatia, Dianne Sylvester, Bhavna Padhye, Caroline Bateman, David Lord, Geoffrey McCowage, Luciano Dalla-Pozza, Timothy Harrington, Brendan Steinfort, Mark Dexter, Sarah Josephi-Taylor, Russell Dale, Smadar Kahana-Edwin

**Affiliations:** 1 The Children’s Hospital at Westmead Clinical School, Faculty of Medicine and Health, The University of Sydney, Westmead, NSW Australia; 2 Department of Medical Imaging, The Children’s Hospital at Westmead, Westmead, NSW Australia; 3 Children’s Cancer Research Unit, The Children’s Hospital at Westmead, Westmead, NSW Australia; 4 Department of Oncology, The Children’s Hospital at Westmead, Westmead, NSW Australia; 5 Department of Neurosurgery, Westmead Hospital, Westmead, NSW Australia; 6 Department of Clinical Genetics, The Children’s Hospital at Westmead, Westmead, NSW Australia

**Keywords:** Peripheral vascular disease, Personalized medicine

## Abstract

**Background:**

Arterio-venous malformations (AVMs) have been associated with somatic genetic variants in the RAS-MEK pathway, generating interest in the role of MEK inhibitors. However, open biopsy for molecular characterisation carries a potentially life-threatening bleeding risk.

**Methods:**

We utilized liquid biopsy from the AVM efferent draining vein in 10 children and young adults (female 7, age range 7.3-22.2 years) with extracranial AVMs to identify the underlying somatic variants.

**Results:**

Here we show identification of somatic mosaic variants in 8 of 10 patients. There were no procedural complications. Two patients with spinal arteriovenous metameric syndrome (Cobb syndrome) demonstrated somatic activating *KRAS* variants. Two additional patients had somatic in-frame deletion-insertion variants of *HRAS*. Three patients had other variants involving the RAS-MEK pathway and one within *PIK3CA*. Five patients have commenced molecularly directed pharmacotherapy leading to reduced disability. Variant allele frequency was inversely correlated with sampling distance from the nidus. One patient who underwent sampling at two separate embolization sessions demonstrated a higher variant allele frequency in the sample obtained after embolization was completed.

**Conclusions:**

Our study further supports the role of the RAS-MEK pathway in AVM pathophysiology. In addition, we demonstrate the effective use of liquid biopsy to genotype extracranial AVMs in children. Finally, we provide insights into how the localized high-pressure high-flow conditions within the AVM distinctly shape cell free DNA fragmentation patterns.

## Plain language summary

Arterio-venous malformations (AVMs) are abnormal clusters of blood vessels with fast flow that cause marked disability and most affect children and young adults. There is growing evidence that AVMs may be due to local genetic changes in the cells that line the vessel walls of the AVM. However, doing a surgical biopsy carries a high bleeding risk. We researched the use of liquid biopsy to find these genetic changes – which involves taking a blood sample from the draining vein of the AVM. We were able to find the genetic changes in 8 out of 10 children and young adults in this study. Five of these patients have since been able to start special targeted medicines designed to block the effect of those genetic changes.

## Abstract (web summary)

Bhatia et al. evaluate the ability of draining vein liquid biopsy to identify RAS-MEK pathway somatic genetic variants within children and young adults with extracranial arterio-venous malformations (AVMs). They demonstrate the effectiveness of using liquid biopsy to genotype extracranial AVMs in children.

## Introduction

Arterio-venous malformations (AVMs) are high-flow vascular anomalies characterized by abnormal communications between arteries and veins without a normal intervening capillary network^1^. Extracranial AVMs are typically congenital vascular malformations arising from errors during vasculogenesis^2^. They have an estimated incidence of approximately 1 per 100,000 person-years and the majority involve the head and neck region^3^, though this reported distribution may reflect a publication bias towards lesions that are often surgically resected. A minority of AVMs are associated with germline genetic variants, such as in Hereditary Haemorrhagic Telangiectasia (HHT) or Capillary Malformation-AVM (CM-AVM) Syndrome^2^. However, the majority are sporadic lesions thought to arise from somatic mosaic genetic variants affecting vascular endothelial cells in-utero^2^. Somatic variants in the RAS-MEK pathway, a transcription pathway that controls cellular differentiation and proliferation, are particularly implicated in AVMs^4,5^.

As extracranial AVMs expand over time with increasing arterio-venous shunting of blood away from adjacent tissues and into the venous system, they cause pain and deformity in affected children and young adults, before progressing towards tissue destruction^6^. Involvement of the head and neck region has additional functional impacts on vision, hearing, and swallowing, as well as the psychological impacts of cosmetic deformity in this vulnerable population^3,6,7^. Thus, extracranial AVMs represent a progressive disabling pathology affecting young people with lifelong consequences.

Small, compact extracranial AVMs can be treated with endovascular embolization, surgical resection, or even radiosurgery^3,6,8^. However, large complex AVMs or those involving high-risk locations (such as the orbit, airway, spine, genitalia, or digits) are difficult to manage and often cannot be cured despite multimodal approaches. Incomplete resection or embolization is associated with a high recurrence rate^9^. Therefore, new approaches are needed to manage these complex lesions that cause progressive disability.

Given the implicated role of somatic genetic variants of the RAS-MEK pathway in AVM pathogenesis, there is increasing interest in the potential of molecularly targeted pharmacotherapy to reduce disability^10–12^. However, the existing evidence base for the off-label use of MEK inhibitors (such as trametinib – originally designed for the treatment of BRAF-mutated melanoma) in these lesions is predominantly limited to case reports amongst patients with a confirmed genetic diagnosis^10,11^. In addition, MEK inhibitors carry a significant side-effect profile including diarrhoea, fatigue, oedema, and rash/dermatitis^13^. Thus, targeted pharmacotherapy with MEK inhibitors to treat extracranial AVMs ideally requires a confirmed molecular diagnosis.

Sample procurement to confirm molecular diagnosis is challenging in complex AVMs. Biopsy of large or complex extracranial AVMs carries a high risk of bleeding, which may be life-threatening^14^. A biopsy may also carry an unacceptable risk of injury to the adjacent eye, airway, genitalia, or digits. For these reasons, core tissue biopsy is typically avoided in such patients, impeding the potential to genotype the lesion and offer targeted pharmacotherapy.

An alternative emerging approach to obtain somatic material is liquid biopsy^5^. Liquid biopsy involves acquiring patient’s blood, which may carry low levels of circulating cell-free DNA (cfDNA) that has been shed from degraded cells within the lesion of interest (in this case, vascular endothelial cells)^15^. The first broad application of cfDNA analysis was for non-invasive prenatal testing to screen for foetal chromosomal anomalies during pregnancy. In oncology, liquid biopsy is increasingly utilized as a minimally invasive method to assign targeted treatment and monitor microscopic disease burden during chemotherapy^16^.

Liquid biopsy has recently been adapted to the setting of extracranial AVMs. Palmieri et al.^5^, successfully obtained liquid biopsies from five adult patients with extracranial AVMs by aspirating blood through a catheter placed by transvenous endovascular approach into the draining vein of the AVM^5^. They identified pathogenic activating variants at low frequency involving the *KRAS* gene (a component of the RAS-MEK pathway; variants G12D and G12V) in all five patients with higher variant allele frequency (VAF) in the samples obtained from the AVM primary draining vein compared with peripheral blood samples^5^. This small but important study provided proof-of-concept that liquid biopsy could be utilized to genotype AVMs, with the same group subsequently reporting similar findings in an expanded cohort of 11 patients with extracranial AVMs (*KRAS* and *MAP2K1* somatic variants)^17^. These results have more recently been reproduced by El Sissy et al.^18^, in a larger study of 55 adult patients with extracranial AVMs with a diagnostic yield of 23.6% for identifying pathogenic variants^18^.

Based on the promising early results of Palmieri et al and El Sissy et al, we aimed to demonstrate the utility of liquid biopsy for somatic genotyping of AVMs in children and young adults, with a view to potentially offering targeted pharmacotherapy for selected patients with disabling symptoms. Included patients had complex high-flow vascular malformations for which endovascular treatment or diagnostic catheter angiography was being undertaken. Paired liquid biopsy samples were obtained from the draining vein of the AVM and from peripheral venous blood for a subset of patients.

Our results, in summary, demonstrate successful identification of pathogenic somatic variants along the RAS-MEK pathway using liquid biopsy in 8 of 10 patients with extracranial AVMs. As a result, five patients are able to commence targeted pharmacotherapy, with all five demonstrating improvement in their functional status.

## Methods

### Study design and enrolment/consent

This was a prospective single-arm cohort study of paediatric and young adult patients (all of age <18 years at time of initial diagnosis and treatment) with arterio-venous malformations (AVMs) causing disabling symptoms despite endovascular +/− surgical therapy. The primary aim was to assess for the presence of somatic mosaic variants involving the RAS-MEK and PI3K-mTOR pathways in paediatric patients with AVMs. The secondary aim was to further confirm the feasibility of transvenous liquid biopsy of blood from the efferent draining vein of AVMs as a method of assessing for somatic mosaic variants via cfDNA extraction.

### Inclusion and exclusion criteria

Eligible patients were of age 18 years or less at the time of their initial AVM diagnosis but remained eligible if their actual liquid biopsy/embolization procedure occurred prior to age 25 years and had previously been assessed within the Sydney Children’s Hospital Network. The diagnosis of extracranial AVM required imaging confirmation by catheter-based digital subtraction angiography. Patient undergoing liquid biopsy during an embolization procedure required clinical eligibility and separate clinical consent for the embolization procedure. Patients with pure intracranial AVMs were excluded from the study.

### Sample collection and processing

AVM efferent draining vein samples were obtained by aspiration from a microcatheter (0.018-0.027in internal diameter) or guide catheter (4-5 Fr) placed by endovascular transvenous approach during embolization or angiographic procedures performed under general anaesthesia. Paired peripheral vein samples were collected during the same anaesthesia, typically at the time of venous sheath placement into the femoral vein. For small compact AVMs or intra-osseous AVMs in which direct nidal puncture was being undertaken for embolization, the blood sample was obtained directly from the percutaneous puncture needle. Each sample consisted of 3-5 mL of blood. Samples were collected in Streck tubes (Cell-Free DNA BCT®, STRECK, La Vista, NE, USA, Catalogue No. 218997) and processed for plasma at ambient temperature in a double-centrifugation protocol as previously described^19^. Plasma aliquots were stored at −80 °C until DNA isolation.

### DNA isolation and quantification

Cell-free DNA (cfDNA) was extracted from 1 to 4 mL of frozen plasma samples using the QIAamp Circulating Nucleic Acid kit (Qiagen, Hilden, Germany, Catalogue No. 55114) according to the manufacturer’s instructions, except for increasing the proteinase digest step to 60 min for plasma samples collected in Streck tubes, as recommended by the Streck product literature. DNA was eluted in the 50 µL buffer provided with the kit and stored at −80 °C until analysis. Genomic/constitutional DNA was extracted from PBMC using QIAGEN DNeasy Blood & Tissue kit (Qiagen, Hilden, Germany, Catalogue No. 69504) according to the manufacturer’s instructions. Genomic DNA was extracted from an archived FFPE lesion slices using QIAamp DNA FFPE Tissue Kit (Qiagen, Hilden, Germany, Catalogue no. 56404) according to the manufacturer’s instructions. DNA was quantified using the Qubit dsDNA High Sensitivity Assay Kit for the Qubit 2.0 Fluorometer (Life Technologies, Carlsbad, CA, USA).

### Massive parallel sequencing (MPS) library preparation and sequencing

Dual-indexed, error-correcting 8-bp unique molecular identifiers (UMI) barcoded libraries were prepared using xGen™ cfDNA & FFPE DNA Library prep kit (IDT, Coralville, IA) followed by a custom xGen™ hybridization capture for MPS target enrichment (IDT, Coralville, IA). Probes were designed to cover both strands of the DNA, in a dense 2x tailing. The panel provides 100% coverage of all coding exons plus 10 bases of flanking non-coding DNA of the genes: *KRAS, HRAS, NRAS, RASA1, BRAF, MAP2K1, MAP2K2, EPHB4, ACVRL1, SMAD4, ENG, GNAQ, PTEN, PIK3CA, TEK, and MAP3K3*. CfDNA input amount ranged between 14.3 to 25.3 ng (mean 22.0 ng). Ultra-deep sequencing of the libraries was carried out on Illumina NovaSeq platform to generate 2x150bp reads at a raw coverage of 44,152-164,203x (mean 98,110x) at the Australian Genome Research Facility (AGRF), Australia.

For PBMC genomic DNA, 100 ng was fragmented to a target size of 200 bp using a Bioruptor sonicator and sequenced at a raw coverage of 151,939-267,282× (mean: 214,729×). Limit of detection (LoD) and limit of blank (LoB) were evaluated with 20 ng of commercial artificial cfDNA reference standards that include the variants: KRAS:p.G12D, NRAS:p.Q61K, NRAS:p.A59T, PIK3CA:p.E545K at 1%, 0.1%, 0.05%, and 0% frequencies (Horizon Discovery, Multiplex I cfDNA Reference Standard Set, Catalogue No. HD780) [0.05% standard was prepared in-house using equal amounts of the 0.1% and 0% standards]. Under this framework, LoD of 0.05% and LoB of 0% (no false positives) were determined (Supplementary Fig. S4).

### Sequencing data analysis and variant calling

Sequence data was processed using the error correction method by collapsing combined read families. UMI extraction, read alignment, error correction, read grouping, consensus read processing, and variant calling were performed using BWA-mem v0.7.17, Picard v2.21.9, fgbio v0.8.0, and VarDict v1.8.2. VCF files were subsequently annotated with ANNOVAR. Variants were filtered on quality and prioritization based on rarity in population datasets and function annotation such as nonsynonymous, exonic and loss of function. Genomic regions with identified variants were manually reviewed with Integrative Genomic Viewer v2.13.2.

### cfDNA fragment size analysis

To establish the size distribution of cfDNA molecules, we queried for cfDNA molecules overlapping the genomic positions of the identified variants. We extracted the cfDNA fragment size of wildtype and variant reads with length <800 bp. The distribution of fragment length was calculated and compared by dividing the number of fragments by the total number of fragments.

### Orthogonal confirmation

ddPCR – NM_004985(KRAS):c.35 G > A (p.G12D) [KRAS p.G12D] was analysed using PrimePCR variant detection assay dHsaMDV2510596 (KRAS:c.35 G > A, p.G12D, 6-FAM, KRAS WT reference gene, HEX; Bio-Rad Laboratories) as described in Kahana-Edwin et al., 2021^19^. Controls included a non-template control (NTC), which contained purified water instead of cfDNA, and the artificial cfDNA reference standards described above (Horizon Discovery). The ddPCR reaction mixture was used for droplet generation, and amplification was carried out in a C1000 Touch Thermal Cycler (Bio-Rad Laboratories) under the following conditions: 95 °C for 10 min, 40 cycles of 94 °C for 30 s, 55 °C for 1 min; then 98 °C for 10 min. ddPCR was performed using the QX200 ddPCR system according to the manufacturer’s instructions (Bio-Rad Laboratories). QuantaSoft™ Analysis Pro v1.0 software (Bio-Rad Laboratories) was used for data analysis. Target and reference/s copies were within the dynamic range of the instrument to ensure accurate detection level.

FFPE genomic DNA underwent MPS library preparation and sequencing as described above.

### Statistics and reproducibility

Statistical analyses (Pearson’s linear correlation, unpaired t-test, Mann-Whitney test, multiple unpaired t-test, box-plot, histogram, and Gaussian distributions) were undertaken using GraphPad Prism version 10.2.3.

### Commencement of targeted pharmacotherapy

Patients with identified pathogenic somatic variants were referred to the paediatric oncology service at our institution and were assessed by one of the four oncologists involved in the study (BP, CB, GM, LD-P). Dependent on the level of functional disability, off-label use of targeted pharmacotherapy was offered on a compassionate basis to patients for the purposes of symptom reduction. Such compassionate use of MEK pathway inhibitors required separate Sydney Children’s Hospital Network drug committee approval (occurring outside the setting of a clinical trial) and provision of genotyping results (with the participant’s permission) to the supplying pharmaceutical company.

### Ethics

Ethics approval for this study was granted by the Sydney Children’s Hospital Network Human Research Ethics Committee (2021/ETH12371). Informed written consent to participate in the study was obtained from the patient or their primary caregiver for all participants. All study procedures were undertaken according to principles of the Declaration of Helsinki.

### Reporting summary

Further information on research design is available in the Nature Portfolio Reporting Summary linked to this article.

## Results

### Patient characteristics

The cohort included 15 samples of liquid biopsy in 10 patients (female 7, male 3) with extracranial AVMs who had a mean age of 12.9 years (SD 4.76, range 7.3–22.2 years) at the time of biopsy. The presence of 15 samples was accounted for by the collection of two separate draining vein samples each from two patients at separate treatment sessions, three patients having additional peripheral blood testing, and one patient having only a peripheral blood sample tested. The anatomical locations of AVM involvement included the face (*n* = 4), fingers (2), pelvis (1), lower limb (1), and spinal cord with additional involvement of the overlying metameric skin and paraspinal tissues (2: spinal arteriovenous metameric syndrome – SAMS; formerly termed Cobb syndrome). All 10 patients had previously undergone endovascular treatment(s) without successful angiographic or clinical cure and had persistent symptoms or disability relating to their condition. Patient characteristics, AVM locations, staging, liquid biopsy sites and genetic findings are summarized in Table 1.

**Table 1:** Patient characteristics, AVM phenotype, and genotype results from liquid biopsy samples

Patient	Sex (F/M)	Clinical phenotype	AVM location	AVM Staging (SECg)^27^	AVM Staging (Schobinger)^28^	MPS findings: Variant	Liquid biopsy session	Age range (years) at time of LB	AVM nidus maximal diameter MRI (mm)	Draining vein sample VAF %; Distance from nidus	Peripheral vein sample VAF %; timing relative to embolization	PBMC MPS findings VAF %	Orthogonal testing VAF % (95% CI)
**1**	F	Orbital and nasal AVM	Face	S3 E2 C2 g+	Stage III	*KRAS* G12D	1	19–25	124	3.1% 10 mm	NA	0%	ddPCR of draining vein sample2.58% (0.36%)
**2**	F	Pelvic, perineal, gluteal and upper thigh AVM with pelvic soft tissue overgrowth	Pelvis	S3 E3 C2 g+	Stage III	*HRAS* T58_G60delinsVLDVL	1	9–12	151	0.5% 15 mm	NA	NT	Surgical biopsy tissue: MPS, 5.0%
							2	9–12		20.1% 0 mm (nidal^a^)	6.1% 1 hour post		
**3**	M	Spinal AV metameric syndrome	Cervical spine: SAMS	S4 E2 C2 g+	Stage III	*KRAS* G12D; *BRAF* K601E	1	13–18	141	NA	0.14%; 0.07%	0%; 0.28%	ddPCR 0.12% (0.08%); NA
**4**	M	Finger AVM: middle phalanx	Finger	S2 E2 C1 g-	Stage II	*MAP2K1* K57N	1	13–18	29	0.09% 10 mm	0% 1 hour prior	0%	NA
**5**	F	Finger AVM:Middle phalanx	Finger	S2 E2 C1 g+	Stage II	*HRAS* A59_E62delinsWPA	1	3–8	18	3.1% 0 mm (nidal^a^)	NA	NA	NA
							2	3–8		2.3% 0 mm (nidal^a^)	NA		
**6**	M	Buccal and peri-maxillary AVM	Face	S2 E3 C1 g+	Stage III	*MAP2K1* K57N	1	9–12	49	0.13%15 mm	1. 1 hour post	0%	NA
**7**	F	Complex orbital lymphovascular malformation with AVM component	Face	S3 E2 C2 g+	Stage II	*PIK3CA* H419_C420delinsQR	1	3–8	19	3.3% 5 mm	NA	0%	NA
**8**	F	Peri-maxillary and nasal AVM	Face	S3 E2 C2 g+	Stage III	None identified	1	3–8	49	None identified	NA	NT	NA
**9**	F	Posterior thigh AVM	Lower limb	S1 E2 C1 g-	Stage II	None identified	1	13–18	58	None identified	NA	NT	NA
**10**	F	Spinal AV metameric syndrome	Thoracic spine: SAMS	S4 E2 C2 g+	Stage III	*KRAS* Q61H	1	19–25	214	0.9% 50 mm	NA	0%	NA

*AV* arteriovenous, *AVM* arteriovenous malformation, *CI* confidence interval, *ddPCR* digital droplet PCR, *NA* not available, *NT* not tested, *F* female, *LB* liquid biopsy, *M* male, *MRI* magnetic resonance imaging, *MPS* massive parallel sequencing, *PBMC* peripheral blood mononuclear cells, *SAMS* spinal arteriovenous metameric syndrome, *VAF* variant allele frequency.

^a^Sample obtained via direct access to the AVM nidus during percutaneous direct puncture embolization.

### Technical success of liquid biopsy acquisition

**Fig. 1:**
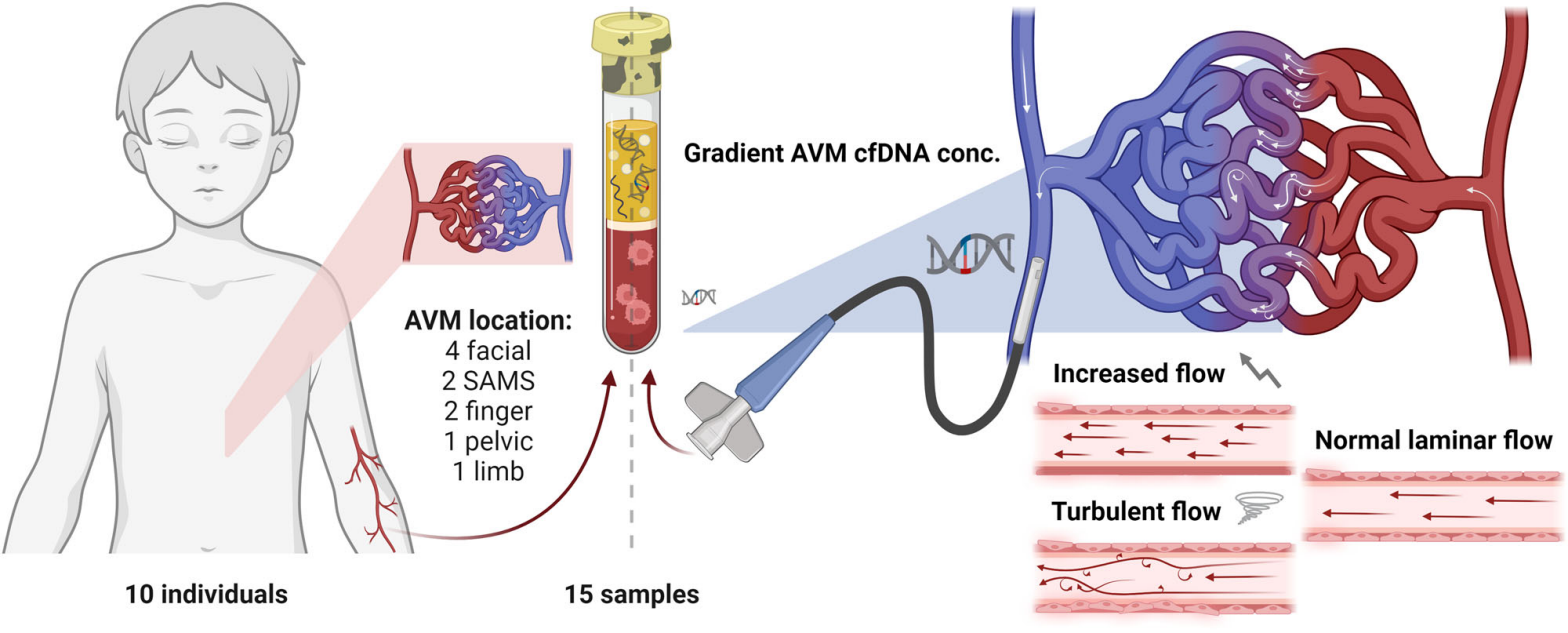
Liquid biopsy technique for AVMs. Overview of the cohort and liquid biopsy samples utilized in this study. Diagrammatic representation of the patient cohort and the AVM anatomical locations is provided on the left. On the right, a schematic diagram of an AVM shows arterial inflow (red) connecting via a complex central nidus to the draining efferent vein (blue). Turbulent flow within the nidus is hypothesised to promote shedding of endothelial cell- derived cfDNA into the circulation. A microcatheter, placed via a transvenous approach, is positioned in the draining efferent vein to sample AVM-enriched cfDNA from the blood. The aspirated blood is transferred into a specialised cfDNA collection tube for downstream analysis. Created in http://BioRender.com.

Endovascular liquid biopsy from the draining vein of the AVM was successfully obtained from all 9 patients in whom this was attempted – see Fig. 1. In two of these patients, additional draining vein samples were successfully obtained at a separate second session of endovascular treatment. There were no periprocedural complications related to liquid biopsy and all obtained samples yielded cfDNA material that underwent massive parallel sequencing (MPS) using our bespoke vascular malformations gene panel (see Methods). In addition, synchronous peripheral venous blood samples were collected for 3 patients.

In one patient with cervical SAMS causing progressive paraparesis (Patient 3 in Table 1), only peripheral venous blood was obtained as it was determined at a multi-disciplinary team meeting that there were no further safe endovascular treatment options. Because of the patient’s progressive disability, it was determined that peripheral vein liquid biopsy alone should be undertaken in the first instance.

### Liquid biopsy genotyping

The genotype results are detailed in Table 1. Pathogenic or likely-pathogenic somatic mosaic variants were identified in 8 out of the 10 included patients, including in one patient with only a peripheral vein blood sample (Patient 3). This sample finding was orthogonally confirmed on digital droplet PCR secondary testing (Supplementary Fig. S1).

To mitigate clonal haematopoiesis (CH)-related false positives, we genotyped matched genomic DNA from peripheral blood mononuclear cells (PBMC) in 6 of the 8 variant-positive cases. Notably, in Patient 3, both *KRAS* and *BRAF* variants were detected in cfDNA, but the same *BRAF* variant was also identified at a slightly higher VAF in PBMC genomic DNA, suggesting a CH-related origin rather than AVM association. In contrast, all other variants were absent in PBMC genomic DNA, supporting their AVM origin. Among the remaining two variant-positive cases, one (Patient 2) was verified in an archival FFPE surgical biopsy, while the other lacked available PBMC material for confirmation.

**Fig. 2:**
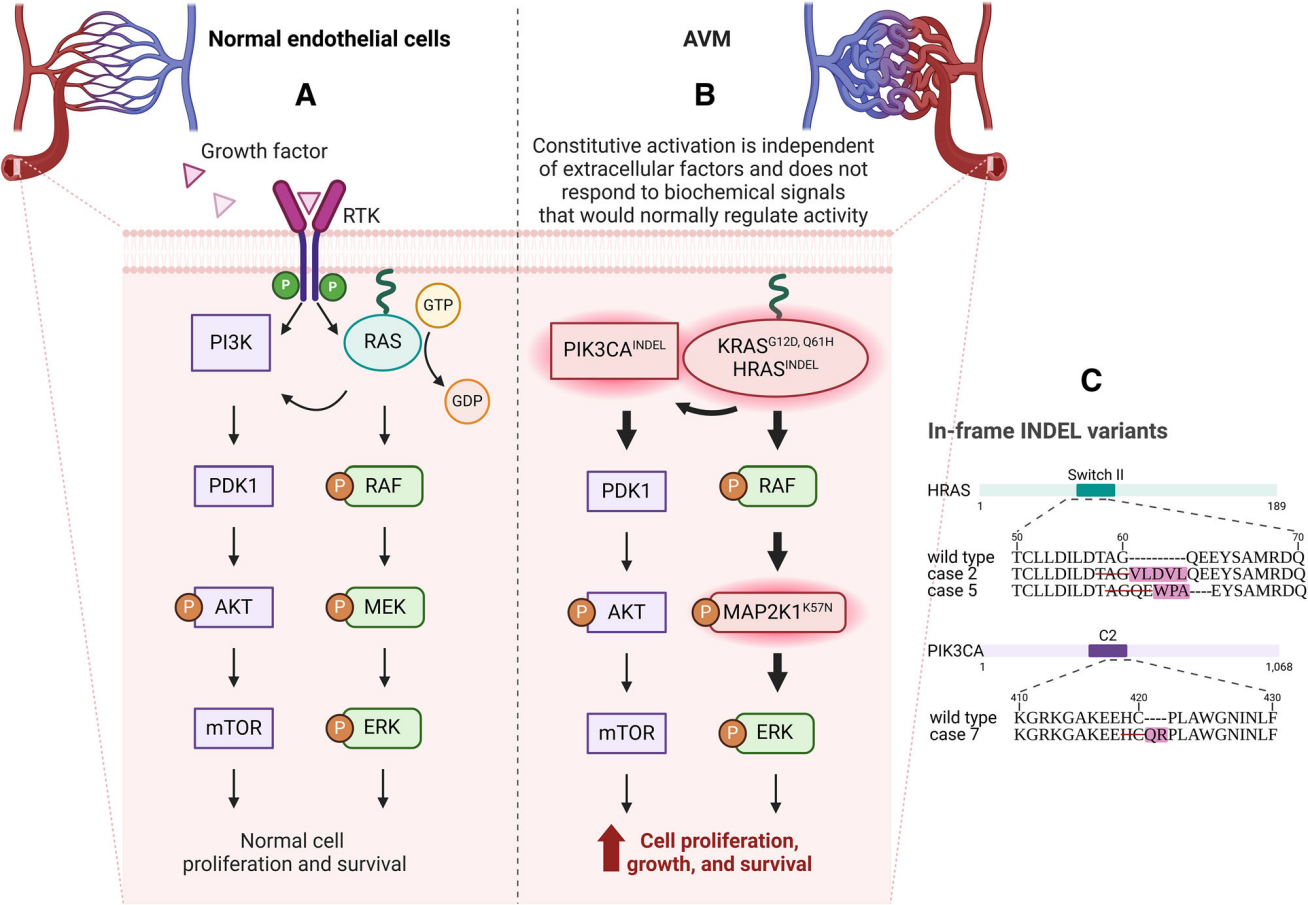
The RAS-MEK and PI3K-mTOR signalling pathways in normal endothelial cells and AVMs. **A** Schematic of normal signalling in endothelial cells. Growth factors bind to the extracellular domain of the receptor tyrosine kinases (RTKs), activating PI3K (purple) and RAS GTPase (aqua). Note that RAS activation also upregulates PI3K function – highlighting a key point of crosstalk between the two pathways. **B** Hypothesized dysregulation in AVM endothelial cells, characterized by activating pathogenic variants in *KRAS*, *HRAS*, *MAP2K1*, and *PIK3CA* identified in this study. These variants result in constitutive pathway activation, promoting increased proliferation, survival, and angiogenesis. **C** Domain maps of *HRAS* and *PIK3CA* affected by the in-frame indels, with amino acid sequence alignment. Deletions are indicated by strikethroughs, and the novel sequences are highlighted in light purple. Created in http://BioRender.com.

The identified pathogenic or likely-pathogenic variants involved the classic hotspot activating point variants in *KRAS* (3 patients: G12D, G12D, Q61H) and *MAP2K1* (2 patients: K57N, K57N), as well as in-frame insertions-deletions (indels) in *HRAS* (2 patients: p.Thr58_Gly60 and p.Ala59_Glu62) and *PIK3CA* (1 patient: p.His419_Cys420) – see Table 1 and Fig. 2. Of the 8 variants identified, 7 involved the RAS-MEK pathway (*KRAS*, *MAP2K1*, *HRAS*) and one involved the PIK3CA-mTOR pathway (*PIK3CA*), the latter of which is more often implicated in low-flow vascular malformations; see Fig. 2.

Both patients with SAMS (Patients 3 and 10) had *KRAS* activating missense variants (G12D and Q61H respectively).

### Variant allele frequency (VAF) correlates with distance from nidus

The sampling point for liquid biopsies from the draining vein of AVMs varied between patients – see Table 1. In two patients (Patients 2 and 5), samples were obtained during direct puncture of the nidus – the complex junction point between abnormal arteries and veins forming the central core of the AVM. In other patients, the sampling point varied in distance from the nidus by 5 – 50 mm.

**Fig. 3:**
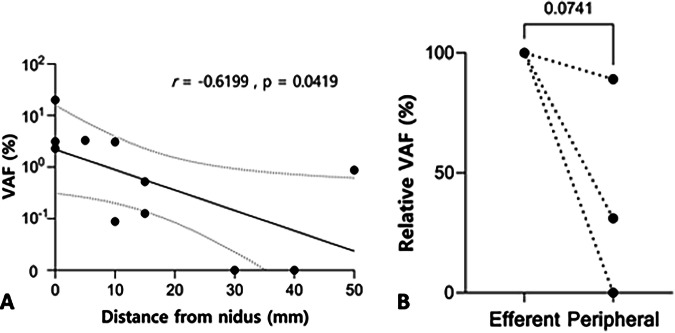
Correlation between cfDNA VAF and blood draw distance from the AVM nidus. **A** Linear regression and 95% confidence bands of the regression line (dotted lines) of cfDNA VAF (log_10_-transformed) and draw point distance from the AVM nidus (*n* = 11 samples from 9 patients). Zero values were replaced with 0.0001 prior to log transformation to allow plotting on the logarithmic scale. Pearson coefficient *r* = −06198, *p* = 0.0420; **B** Correlation between relative cfDNA VAFs in matched plasma samples collected from peripheral veins and efferent veins in three patients (*n* = 3 paired sample sets). One-tailed paired *t*-test *p* = 0.0741.

Linear correlation analysis of the VAF in efferent draining veins demonstrated that VAF decreased with increasing sampling distance from the nidus (mm) (Pearson’s correlation test, *r* = −0.6198, *p* = 0.0420 – see Fig. 3A). The variant concentration also demonstrated a significant negative correlation with the sampling distance from the nidus (see Supplementary Fig. S2), but the small sample size limits strong conclusions. Similarly, VAF was reduced by 11-100% in all synchronous peripheral blood samples (available for 3 patients) but without a significant correlation, most likely due to the small sample size (one-tailed paired t-test *p* = 0.0741 – see Fig. 3B). No significant correlation was identified between AVM nidus volume, as measured on MRI, and VAF (see Figure S3, Table 1).

### Fragment size profiling and spatial variations in AVM-derived cfDNA

**Fig. 4:**
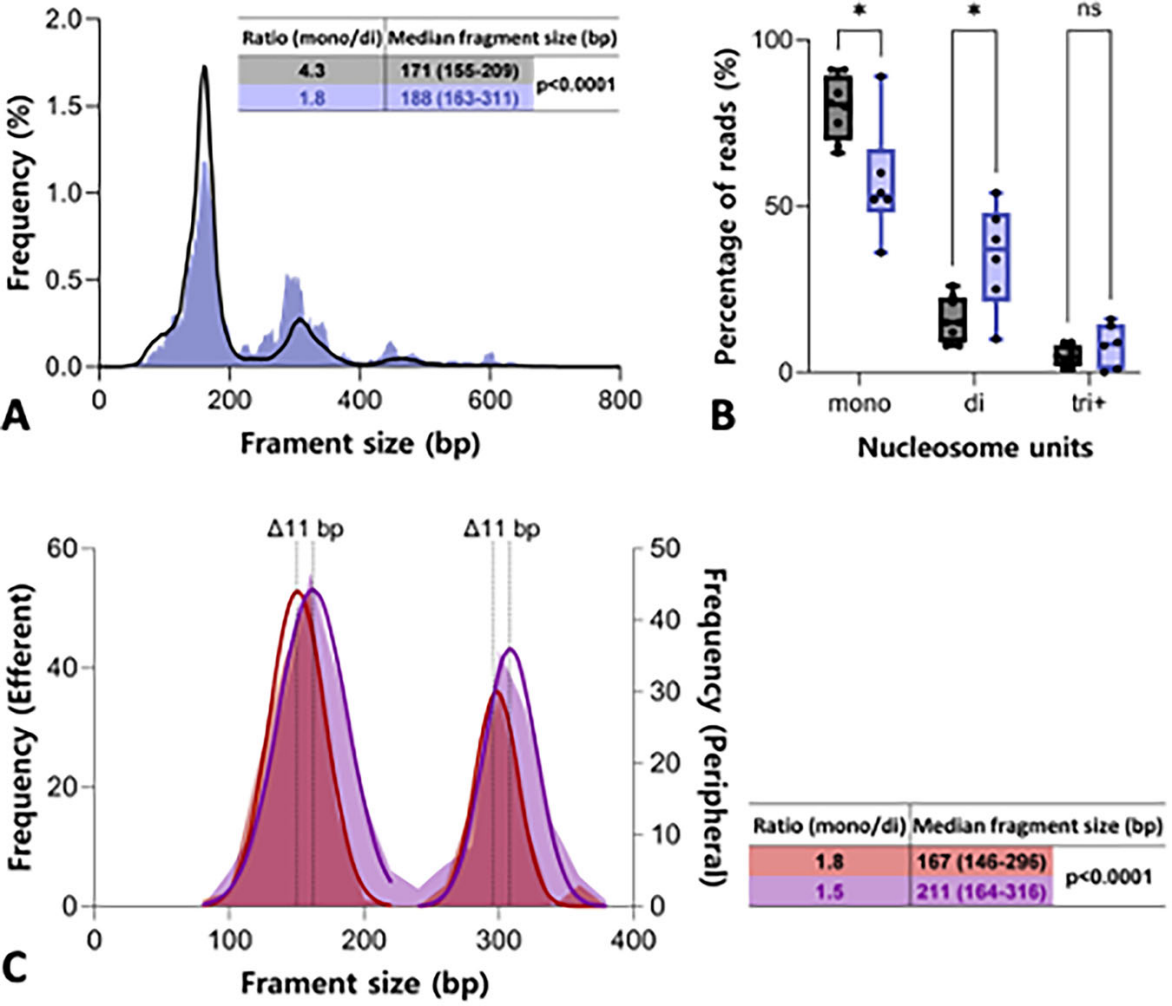
Size profile of cfDNA reveals nucleosome pattern and dynamics. **A** Fragment size distributions of variants (purple) and corresponding wild type (black) reads (*n* = 9). The ratio of the number of reads within the mono-nucleosome (0–240 bp) to di-nucleosome (241–400 bp) peaks is shown. Median fragment sizes across the 0-800 bp range are shown with interquartile range (IQR), and differences were analysed using the Mann-Whitney test. **B** Percentage of reads within different nucleosome units (mono-, di-, and ≥tri-nucleosomes, ≤240 bp, 241–400 bp, and ≥400 bp, respectively) for each sample. Data for variants (purple, only samples with >10 variant reads, *n* = 6) and wild type (grey, *n* = 8) are shown. Statistical significance was assessed by multiple unpaired t-test, adjusted *p* = 0.028 (indicated by an asterisk). ‘ns’ denotes non-significant difference (*p* = 0.255). **C** Histogram displaying the distribution of cfDNA fragment sizes for the variant in matched efferent (purple) and peripheral (red) samples (*n* = 1). Gaussian distributions were fitted to the data around the mono-nucleosome peak (R² = 0.9862 for efferent, R² = 0.9650 for peripheral) and the di-nucleosome peak (R² = 0.9378 for efferent, R^2^ = 0.9859 for peripheral), as shown by the overlaid line graphs. The median fragment sizes for the mono-nucleosome (151 bp for efferent vs. 162 bp for peripheral, *p* < 0.0001) and di-nucleosome (298 bp for efferent vs. 309 bp for peripheral, *p* < 0.0001) gaussian peaks are presented in dotted lines and were compared using unpaired t-test. The nucleosome ratio and median fragments sizes were calculated as in (**A**).

AVM pathophysiology and its interaction with the surrounding high-pressure, high-flow environment may influence the mechanisms of cfDNA release into the bloodstream and mark it with specific fragmentation signatures or features. To investigate whether localized factors at the nidus affect not only the concentration but also the fragmentation patterns of AVM-derived cfDNA, we analysed the fragment size profiles of both variant and wild-type cfDNA molecules overlapping the same genomic positions from efferent samples. Wild-type cfDNA, primarily originating from normal haematologic cells, displayed typical nucleosomal patterns, including a dominant peak at approximately 172 bp and periodic smaller peaks at around 316 bp and 476 bp. In contrast, the variant cfDNA exhibited distinct differences, with a 2.4-fold decrease in the mono- to di-nucleosomal peak ratio and consequently, a median fragment size increase of approximately 17 bp (*p* < 0.0001) compared to wild type cfDNA (Fig. 4A). This pattern was consistent across 5 out of 6 samples that met the threshold of having more than 10 variant reads (*p* = 0.028) (Fig. 4B).

The fragmentation pattern of efferent AVM cfDNA is notably different from that observed in peripheral cfDNA from cancer patients, foetal cfDNA in maternal circulation, and cfDNA from transplanted organs, which tends to be shorter and more fragmented^20^. To further distinguish between systemic effects in the peripheral circulation on cfDNA size distribution and local factors at the AVM nidus, we compared matched efferent and peripheral samples for patient 2. This comparison revealed a shift in nucleosomal peak positioning, with upper-end fragments being approximately 11 bp shorter in the peripheral variant sequences (*p* < 0.0001), while the mono- to di-nucleosomal peak ratios remained similar (Fig. 4C). This finding aligns with the observed trend of shorter cfDNA fragment lengths in peripheral circulation across various contexts, reinforcing the notion that localized AVM conditions distinctly shape cfDNA fragmentation patterns.

### Genotype-directed pharmacotherapy

Given the reasonable safety profile of trametinib and the significant disability complex vascular malformations can cause in paediatric patients, five patients (including two with SAMS) with identified somatic mosaic variants in the RAS-MEK pathway (Patients 1, 2, 3, 6, and 10) have been commenced on genotype-directed pharmacotherapy, with approval by the pharmaceutical companies on compassionate grounds. All five patients have had a marked clinical and functional improvement since commencing pharmacotherapy when measured using the modified Rankin Scale [mRS] (mRS scores improving from 2 to 1 for Patient 1, from 2 to 1 for Patient 2, from 4 to 2 for Patient 3, from 2 to 1 for Patient 6, and from 3 to 2 for Patient 10). However, two of the five patients have had to manage associated dermatological side effects from the medications that subsequently responded to topical steroids and dose adjustment; in particular, rash related to trametinib which reached a maximal severity on the Common Terminology Criteria for Adverse Events (CTCAE) Version 5.0^21^ of Grade 1 for Patient 1 (mild symptoms) and Grade 2 for Patient 3 (moderate symptoms limiting important activities of daily living).

**Fig. 5:**
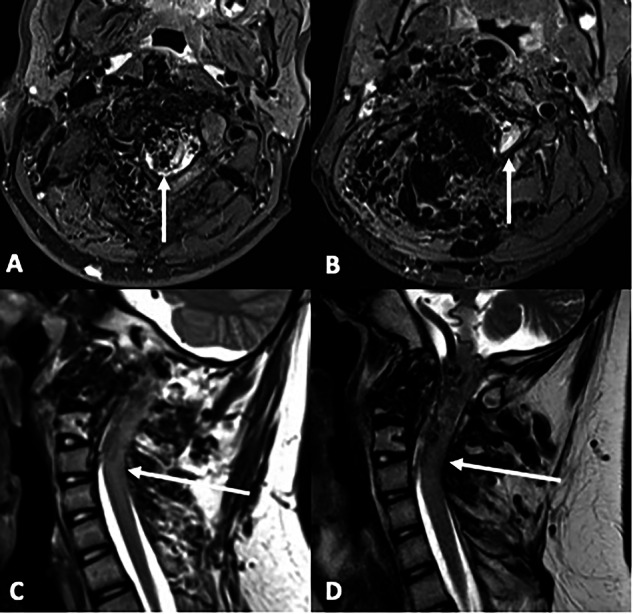
Magnetic resonance imaging in a teenage patient with cervical spinal arteriovenous metameric syndrome (SAMS) with cervical spinal cord AVM. Patient 3 in our study. **A** Axial fat-supressed-T2-weighted-image at the cervico-medullary junction demonstrates an intra-medullary arterio-venous malformation within the spinal cord (arrow). **B** More caudally, the cord is compressed (arrow) by enlarged epidural vessels (which appear black and serpiginous). **C** Sagittal T2-weighted-image (left para-sagittal) shows increased cervical cord signal in keeping with oedema from cord compression and congestion (arrow). **D** After six months of treatment with trametinib, there is a marked reduction in cord signal change/oedema, correlating with improvement in lower limb function. This patient improved from wheelchair dependent paraparesis to walking again.

In Patient 3, who suffered with progressive wheelchair-dependent lower limb paraparesis due to their cervical spine SAMS even after numerous embolization procedures, the clinical response to trametinib resulted in restoration of independent walking and activities of daily living – see Fig. 5.

## Discussion

Our study confirms the presence of somatic mosaic pathogenic variants of the RAS-MEK pathway in a high proportion of extracranial AVMs, including activating variants of *KRAS*, *HRAS*, and *MAP2K1*. In addition, our study further demonstrates the utility and efficacy of liquid biopsy to identify the underlying somatic genetic variants in extracranial AVMs, obviating the need for a high-risk core tissue biopsy to obtain genetic material. There were no complications related to liquid biopsy and the procedure was both acceptable and tolerable to patients and their families. This study was undertaken in children and young adults, who are the population most severely impacted by AVMs, allowing for a real-world application of this technology in the population of greatest need. This approach would allow clinicians to genotype AVMs at a lower bleeding risk and therefore increase potential access to targeted pharmacotherapy for these disabling conditions.

The data presented here support existing literature indicating that most patients with intracranial or extracranial AVMs harbour somatic mosaic pathogenic variants of the RAS-MEK pathway. This creates the potential to treat this patient group using MEK inhibitors^10,11^ (or RAS inhibitors, should they become more readily available), originally designed for the treatment of patients with *BRAF*-variant melanoma^22^. However, to date only four cases of such treatment for extracranial AVMs have been reported^10,11,23^. These include three patients with somatic pathogenic variants: a teenager with an in-frame deletion of *MAP2K1* in the AVM, who initially showed disease progression with sirolimus (mTOR inhibitor) treatment but later experienced a significant and sustained response to trametinib^24^; a teenager with SAMS in whom biopsy material (obtained from a capillary malformation in the overlying skin of the metameric syndrome) demonstrated a *KRAS* variant was exhibited a substantial decrease (>75%) in blood flow to the AVM after 6 months of trametinib therapy^10^; and a child with an AVM of the ear and neck harbouring a *MAP2K1* variant with an excellent response after 6 months of treatment^25^. A fourth patient, a teenager with CM-AVM syndrome and a germline variant of *EPHB4*, was treated with trametinib for high-output cardiac failure secondary to arteriovenous shunting of blood from multiple AVMs in the thigh^11^. Genotyping for the somatic variants was conducted via tissue biopsy of the AVM^10,24,26^ and for the germline condition via peripheral blood^11^.

These exciting outcomes drove the prospective investigation of the use of MEK inhibitors for this indication, currently undertaken in five phase II trials: NCT04258046, NCT05125471, and NCT05983159 in the United States; 2019-003573-26 in Europe; and NCT05983159 in Australia. However, the requirement for biopsy material from the AVM for genotyping or to provide a pathology report in the US and Australia trials, limits the uptake due to the inherent high bleeding risk, which may be life-threatening^14^.

Whilst these preliminary reports have raised much interest in the potential role of targeted pharmacotherapy for the treatment of AVMs, it should be noted that only a select cohort of AVM patients will warrant pharmacotherapy treatment. Conservative management, endovascular embolization, and surgical resection remain the primary treatment options for the majority of AVMs^12^. Staging of an AVM is essential to assist in determining the optimal treatment options. The SECg staging system for extracranial head and neck AVMs takes account of the surgical/anatomical extent (S), the endovascular/angiographic morphology (E), clinical severity (C), and the presence of recent growth (g) to assist in determining the treatment approach^27^. Under this staging system, targeted pharmacotherapy would generally be reserved for more extensive lesions (S3-4) with disabling clinical features (C2-3) that are not amenable to or have had suboptimal response to other treatment modalities^25,27^. Such a decision would generally be undertaken through a multi-disciplinary team assessment including surgical, medical, and interventional radiology teams. For other peripheral AVMs, the four-stage Schobinger clinical classification system is frequently used (Stage I – quiescence with no clinical symptoms, Stage II – enlargement with pulsations, thrill, or bruit, Stage III – destructive tissue changes, Stage IV – congestive cardiac failure)^28^.

The use of liquid biopsy to obtain circulating cfDNA for the somatic genotyping of AVMs has recently been described in a cohort of 55 adult patients with extracranial AVMs by El Sissy et al.^18^, with a yield of 23.6% in identifying pathogenic variants (predominantly in *MAP2K1* and *KRAS*)^18^. Similar to our study, El Sissy et al. demonstrated in a large adult sample that liquid biopsy is a feasible and safe method to genotype AVMs, as well as similarly demonstrating that the sensitivity of the technique increases with the proximity of the sample site to the nidus^18^. Our study findings further validate these results. In addition, our study demonstrates the feasibility and safety of this technique in children - the age group in which the majority of AVMs present, and a vulnerable population that suffers extensive disability from the physical and psychological impacts of AVMs^7^.

SAMS, formerly termed Cobb syndrome, is a congenital metameric vascular malformation affecting the spine, including the spinal cord, the paraspinal tissues, and overlying skin within the same segment (metamere) of the body. Whilst rare, it is a highly disabling condition affecting children and adolescents with progressive pain, neurological deficit, and eventually paraplegia as the spinal cord becomes more affected^29^. The involvement of the spinal cord by AVM(s) poses a significant risk of permanent deficit with any surgical or endovascular approach. Therefore, alternative treatment options are necessary for an otherwise palliative condition. Edwards et al.^10^ reported surgical biopsy of capillary malformation in the overlying skin of a patient with SAMS who had developed cardiac failure due to the extent of arterio-venous shunting^10^. This demonstrated the presence of an in-frame tandem duplication involving *KRAS* at a 5% frequency^10^. The patient responded successfully to treatment with the MEK inhibitor trametinib. Our finding of somatic activating pathogenic *KRAS* variants (G12D; Q61H) in two additional patients with SAMS (Patients 3 and 10) provides further evidence of the role of *KRAS* variants in this condition. In addition, both of our patients also had strong clinical responses to MEK inhibition with trametinib including restoration of independent walking in a teenager who was previously wheelchair dependent - see Fig. 5. Given the rarity of SAMS, the demonstration of activating *KRAS* variants in three separate patients who all demonstrated significant clinical responses to MEK inhibition suggests a central role of *KRAS* in the pathophysiology of SAMS. In addition, it suggests an important potential role for targeted pharmacotherapy in this incurable condition.

Somatic variants in the *HRAS* gene have only recently been linked to the development of AVMs^30–33^, with all eight reported pathogenic variants involving in-frame indels. In this study, similar *HRAS* indel variants were also identified in two AVM patients (Patients 2 and 5; pelvic and finger AVM locations, respectively, Table 1). Patient 2 (pelvic/perineal AVM; codons affected 58-60) also demonstrated surrounding tissue overgrowth as described in the case report by Konczyk et al.^30^ (facial AVM; *HRAS* codons 58-59)^30^. Such overgrowth was also visible in the provided MR images of a case described by Schmidt et al.^32^ (paraspinal intramuscular AVM; *HRAS* codons 172-179)^32^. This may support the hypothesis that *HRAS*-variant AVMs are associated with tissue overgrowth. Interestingly, whilst the spectrum of variants in *KRAS/HRAS* in human cancer typically involves activating missense variants at codons 12, 13, and 61^34^, *KRAS/HRAS* in-frame indels or in-frame duplications have been associated with vascular malformations^26,31^. Importantly, among *KRAS/HRAS* variants, hotspot variants and in-frame indels were associated with different functional phenotypes. Functional studies of these variants in the switch II region of *HRAS* and *KRAS* have shown that the resulting proteins are weakly but constitutively activating a selective signalling process through the MAPK pathway while diminishing the capacity of KRAS/HRAS proteins to modulate downstream signalling and to activate the PI3K/AKT pathway^26,35,36^. This diminished capacity to modulate the PI3K/AKT pathway may have a clinical correlate in our study in Patient 2 (pelvic/perineal AVM with HRAS in-frame indel variant), who had ongoing episodes of pelvic bleeding whilst being treated with an mTOR inhibitor (sirolimus), but had a sustained remission from bleeding episodes after the commencement of a MEK inhibitor (trametinib). These differences in signalling outputs are crucial for selecting the most effective targeted treatment.

In relation to vascular lesions, *PIK3CA* pathogenic somatic variants are more commonly described in low-flow vascular malformations than in AVMs. Patient 7 in our cohort was found to have a *PIK3CA* variant, with a phenotype that included both a lymphatic low-flow malformation and an adjacent high-flow AVM, consistent with the underlying genotype. To the best of our knowledge, this specific variant has not been previously described. This in-frame indel replaced amino acids histidine and cysteine with glutamine and arginine at positions 419 and 420, respectively. This alteration results in H419Q and C420R, the latter being a well-described pathogenic variant.

Liquid biopsy was able to identify the underlying somatic variants in 8 of our 10 patients with extracranial AVMs (7 via the efferent draining vein), with positive findings in 12 of 15 liquid biopsy samples - see Table 1. No complications were encountered related to the liquid biopsy process. These findings demonstrate that liquid biopsy of the efferent draining vein of an extracranial AVM can be done at low risk with a high diagnostic yield. The initial proof-of-concept studies utilizing liquid biopsy of the efferent draining vein in extracranial AVMs in adult patients^5,17^ have therefore been reproduced in our study and in a younger age demographic of children and young adults.

In addition, the peripheral vein blood sample, well separated from the AVM location and likely representing genetic material that has passed from the systemic venous return and across the pulmonary circulation before recirculating, yielded the underlying variant in three of four patients who had their peripheral blood sample sequenced, although with reduced VAF. The lower VAF in peripheral blood samples compared with the AVM draining vein can likely be explained by dilution due to admixing with blood not draining from the AVM. These findings suggest that initial somatic genotype screening of patients with extracranial AVMs could be undertaken via peripheral venous blood collection with a moderate yield. Thus, genetic testing could commence even at the initial outpatient assessment and prior to any embolization or surgical procedures, providing a quick, risk-free, and practical clinical pathway. Such results could then be confirmed by later efferent draining vein sampling as appropriate.

Our study highlights potential challenges in interpreting variants detected exclusively in cfDNA without tissue confirmation. CH is a natural consequence of aging, characterized by the accumulation of somatic mutations and the clonal expansion of haematopoietic stem cells^37^. Notably, some CH-associated mutations occur in genes frequently altered in AVMs, such as *KRAS* and *BRAF*^38,39^. Given that the majority of circulating cfDNA originates from haematopoietic cells^40,41^, CH-related mutations in cfDNA could lead to false-positive detections in AVM liquid biopsy testing, potentially confounding the distinction between biological CH background and AVM-derived cfDNA. While CH has traditionally been associated with aging, advances in ultra-deep NGS have enabled detection in younger individuals, including children^42,43^. In our study, CH was identified in a 16-year-old boy alongside the AVM variant. Alternative explanations for false-positive liquid biopsy results include occult malignancy, however, this is highly unlikely given the low cancer prevalence in our young cohort and the distinct gene drivers typically observed in childhood cancers^44^.

The complex hemodynamic environment within the AVM, characterized by irregular blood pressure, turbulent flow, and increased vascular permeability, likely contributes to greater mechanical shedding of cfDNA. This hypothesis is supported by our findings, which show longer variant cfDNA fragment lengths and higher di-nucleosome proportion compared to non-neoplastic cfDNA. The observed shift towards shorter cfDNA fragments in the matched peripheral blood likely reflects nuclease activity in the systemic circulation, leading to additional fragmentation. This understanding provides insight into how local hemodynamic conditions within the AVM nidus and subsequent systemic factors influence cfDNA dynamics, helping to explain the observed differences in cfDNA fragment sizes in various clinical contexts. Together, these results highlight the intricate relationship between AVM pathophysiology, cfDNA concentration, and fragment size distribution, suggesting that local AVM-specific factors play a critical role in shaping cfDNA characteristics. This may have implications for improving liquid biopsy as a non-invasive tool for both diagnostic and monitoring purposes in AVM management.

A potential confounder that may impact VAF is the timing of sampling relative to embolization. In patient 2 of our study (see Table 1), an efferent vein sample from the AVM during the first procedure obtained prior to commencing embolization had a VAF of 0.5%. At a second procedure on the same patient, a sample was obtained directly from the nidus by percutaneous puncture with a VAF of 20.1% and embolization was then performed. A paired peripheral vein sample was obtained one hour after commencing embolization and demonstrated a VAF of 6.1%. This high concentration in peripheral blood following embolization raises the possibility that embolization itself may increase the shedding of cfDNA from degrading endothelial cells and result in enriched samples. Such enrichment of post-embolization peripheral blood samples compared with pre-embolization samples was also described by Sun et al.^45^ who identified marked increases in variant allele detection rates from peripheral blood samples obtained after embolization of venous malformations in 121 patients, compared with pre-embolization. Our findings would provide further support to the concept of obtaining post-embolization peripheral blood samples to improve diagnostic yield. Based on the results of Sun et al.^45^ and supported by our findings in Patient 2 of post-embolization sample enrichment, we would favour sample collection at the end rather than the start of embolization procedures in future research studies.

This study is impacted by several limitations. Firstly, the sample size is small, and it would be essential to validate the reproducibility of our liquid biopsy methodology in a larger study. Secondly, sequencing of cfDNA from the obtained paired peripheral vein blood samples was only undertaken in four patients due to sample availability. The ability to better correlate the results between the efferent draining vein and peripheral vein samples in all patients would have provided further insights into the diagnostic yield of deep sequencing of cfDNA from peripheral blood samples. Nevertheless, VAF and the sampling distance from the nidus were inversely correlated in our study (see Fig. 3A, B), i.e., obtaining samples close to or from within the nidus resulted in a higher yield of genetic material. These results may therefore impact procedural planning for liquid biopsy, whereby patients whose initial peripheral blood testing did not yield a pathogenic finding could be considered for nidal or peri-nidal blood collection if feasible. The impact of proximity to the nidus and timing of sample acquisition relative to embolization upon diagnostic yield could be assessed in more detail in future studies, perhaps by utilizing multiple collection points (e.g., nidal, draining vein, central vein such as the inferior or superior vena cava, and peripheral venous blood). In addition, such an approach may be useful to further refine the risk profile and health economic analysis of liquid biopsy into the future. Thirdly, the SECg staging system^27^ utilized was designed for extracranial head and neck AVMs rather than peripheral AVMs and as such, we have also provided in Table 1 the Schobinger staging system^28^ results (which was designed as a clinical staging system for all extracranial AVMs). In general, targeted pharmacotherapy is reserved for complex AVMs that cannot be completely surgically resected or occluded by endovascular approaches. The role of targeted therapies in combination with partial resection or occlusion for complex cases warrants future trial investigation.

## Conclusions

 Somatic activating variants of the RAS-MEK pathway are common in extracranial AVMs and can be readily identified using liquid biopsy. We provide further evidence to support the role of *KRAS* pathogenic variants in spinal arteriovenous metameric syndrome (SAMS) and *HRAS* variants in AVMs with tissue overgrowth. Liquid biopsy is a safe, effective, and well-tolerated method to genotype extracranial AVMs in children and young adults. One patient with SAMS had their pathogenic variant identified on a peripheral venous blood sample. Higher variant allele frequency rates were also demonstrated in post-embolization draining vein and peripheral blood samples in another patient. Peripheral venous blood cfDNA analysis warrants further evaluation, particularly in the hours following an embolization procedure.

## Supplementary information

Supplementary Information

Description of Additional Supplementary Files

Supplementary Data

Reporting Summary

## Data Availability

Raw sequencing data have been deposited in the NCBI Sequence Read Archive (SRA) under accession PRJNA1315251. This can be accessed via the following link: https://dataview.ncbi.nlm.nih.gov/object/PRJNA1315251?reviewer=9r3eud2qsgof0rrld2rq0guvdo*.* All other data associated with this study are present in the paper or the Supplementary Materials. The source data for Table 1, Figs. 3a, 4, S2a, 3, and S4 is in Supplementary Data 1 file.
