# Supplementary material for: Harnessing liquid biopsy to unveil RAS-MEK pathway somatic pathogenic variants in extracranial arterio-venous malformations
Source: Commun Med (Lond). 2025 Dec 5;5:508. doi: 10.1038/s43856-025-01174-1 (PMC12680647; doi:10.1038/s43856-025-01174-1)
Supplement: Supplementary file 1 — Supplementary Information [file 43856_2025_1174_MOESM1_ESM.pdf]

SUPPLEMENTARY INFORMATION

for

Harnessing liquid biopsy to unveil RAS-MEK pathway somatic pathogenic variants in extracranial arterio-venous malformations

CONTENTS

PAGE NUMBER	SUPPLEMENTARY CONTENT
**1**	Title page
**2**	Contents
**3**	Supplementary Figure S1:<br>**Verification of *KRAS* G12D variant using ddPCR**
**4**	Supplementary Figure S2:<br>**Correlation between cfDNA variant level and blood draw distance from the AVM nidus**
**5**	Supplementary Figure S3:<br>**VAF levels do not correlate with AVM size**
**6**	Supplementary Figure S4:<br>**LoD and LoB**

Supplementary Figure S1: Verification of *KRAS* G12D variant using ddPCR.

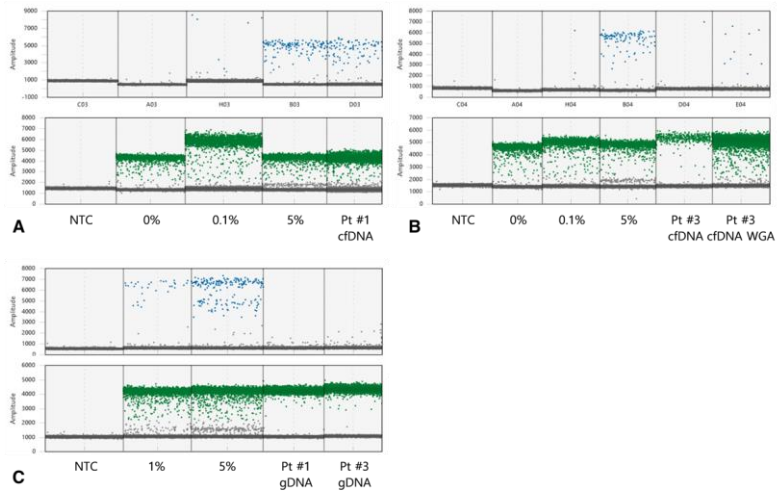

1D fluorescence amplitude plots displaying droplets positive for the *KRAS* G12D variant (blue, FAM channel) and *KRAS* wild type (green, HEX channel). The *KRAS* G12D assay results are shown for cfDNA samples from patients 1 (A) and 3 (B), and genomic DNA from PBMC (gDNA) for both patients (C). These plots demonstrate the presence of the variant exclusively in the cfDNA samples. Positive and negative controls are included, with standards showing variant levels of 0%, 0.1%, 1%, and 5%, as well as NTC (non-template control). cfDNA WGA refers to cfDNA that has undergone whole genome amplification as part of the MPS library preparation.

Supplementary Figure S2: Correlation between cfDNA variant level and blood draw distance from the AVM nidus.

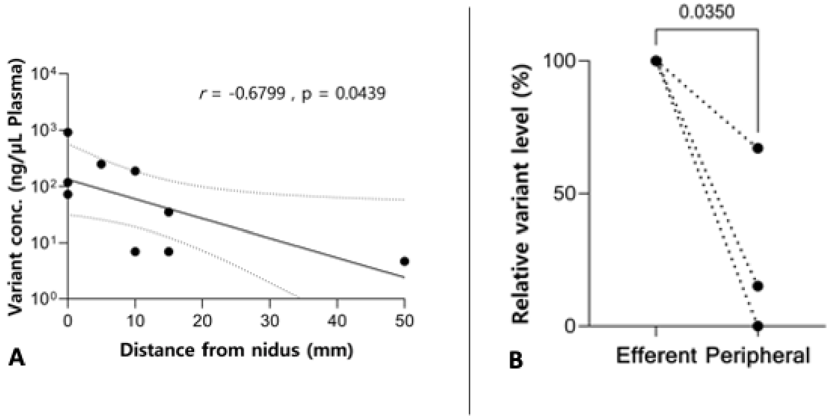

(**a**) Linear regression and 95% confidence bands of the regression line (dotted lines) of cfDNA variant concentrations in plasma and draw point distance from the AVM nidus. Pearson coefficient r=-0.6799, p=0.0439; (**b**) Correlation between the relative cfDNA concentration in plasma samples collected from peripheral veins compared with those from efferent veins. One-tailed paired t-test p=0.0350.

Supplementary Figure S3: VAF levels do not correlate with AVM size.

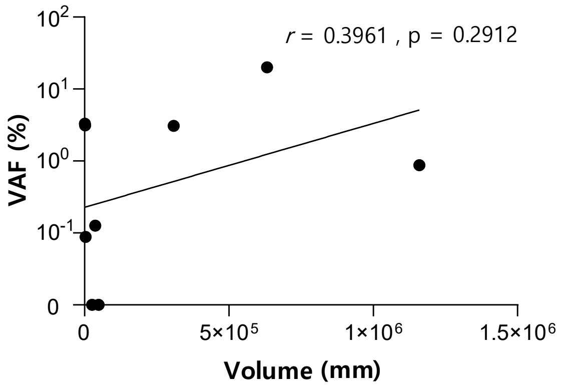

Field Code Changed

Matched AVM volume (assuming an ellipsoid shape) and efferent VAF results from 8 patients. For patients 2 and 5, where two efferent samples were tested, the higher VAF was used. Pearson correlation coefficient is r=0.3961 with p=0.2912.

Supplementary Figure S4: Limit of detection (LoD) and limit of blank (LoB)

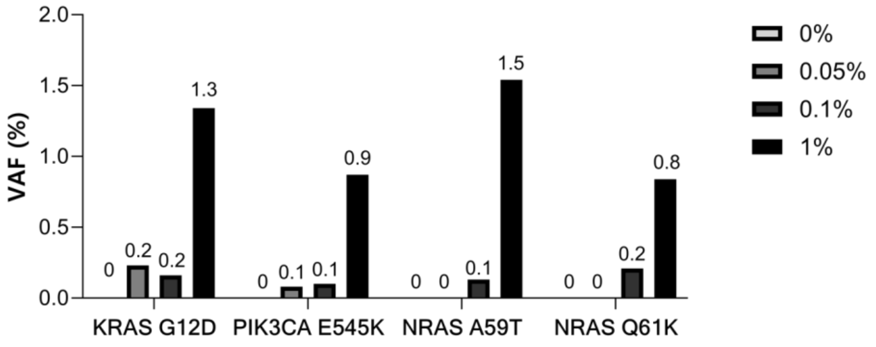

Commercial cfDNA reference standards harbouring the variants: KRAS:p.G12D, NRAS:p.Q61K, NRAS:p.A59T, PIK3CA:p.E545K at 1%, 0.1%, 0.05%, and 0% frequencies were evaluated with the MPS technique. All four variants were detected in the 1%, and 0.1% standards, and two were detected in the 0.05% standard. None of the variants were detected in the 0% standard.
