# Supplementary material for: Harnessing liquid biopsy to unveil RAS-MEK pathway somatic pathogenic variants in extracranial arterio-venous malformations
Source: Commun Med (Lond). 2025 Dec 5;5:508. doi: 10.1038/s43856-025-01174-1 (PMC12680647; doi:10.1038/s43856-025-01174-1)
Supplement: Supplementary file 2 — Description of Additional Supplementary Files [file 43856_2025_1174_MOESM2_ESM.pdf]

Description of Additional Supplementary Files

File name: Supplementary Data

Description: The source data for Table 1, Figures 3a, 4, S2a, 3, and S4
