# Supplementary material for: Harnessing liquid biopsy to unveil RAS-MEK pathway somatic pathogenic variants in extracranial arterio-venous malformations
Source: Commun Med (Lond). 2025 Dec 5;5:508. doi: 10.1038/s43856-025-01174-1 (PMC12680647; doi:10.1038/s43856-025-01174-1)
Supplement: Supplementary file 4 — Reporting Summary [file 43856_2025_1174_MOESM4_ESM.pdf]

nature portfolio

Corresponding author(s): Kartik Bhatia

Last updated by author(s): 28th September 2025

Reporting Summary

Nature Portfolio wishes to improve the reproducibility of the work that we publish. This form provides structure for consistency and transparency in reporting. For further information on Nature Portfolio policies, see our Editorial Policies and the Editorial Policy Checklist.

Statistics

For all statistical analyses, confirm that the following items are present in the figure legend, table legend, main text, or Methods section.

n/a	Confirmed	
☐	☒	The exact sample size (*n*) for each experimental group/condition, given as a discrete number and unit of measurement
☒	☐	A statement on whether measurements were taken from distinct samples or whether the same sample was measured repeatedly
☐	☒	The statistical test(s) used AND whether they are one- or two-sided<br>*Only common tests should be described solely by name; describe more complex techniques in the Methods section.*
☐	☒	A description of all covariates tested
☐	☒	A description of any assumptions or corrections, such as tests of normality and adjustment for multiple comparisons
☐	☒	A full description of the statistical parameters including central tendency (e.g. means) or other basic estimates (e.g. regression coefficient) AND variation (e.g. standard deviation) or associated estimates of uncertainty (e.g. confidence intervals)
☐	☒	For null hypothesis testing, the test statistic (e.g. F, t, r) with confidence intervals, effect sizes, degrees of freedom and P value noted<br>*Give P values as exact values whenever suitable.*
☒	☐	For Bayesian analysis, information on the choice of priors and Markov chain Monte Carlo settings
☒	☐	For hierarchical and complex designs, identification of the appropriate level for tests and full reporting of outcomes
☐	☒	Estimates of effect sizes (e.g. Cohen's *d*, Pearson's *r*), indicating how they were calculated

Our web collection on statistics for biologists contains articles on many of the points above.

Software and code

Policy information about availability of computer code

Data collection	No code used
Data analysis	No code used

For manuscripts utilizing custom algorithms or software that are central to the research but not yet described in published literature, software must be made available to editors and reviewers. We strongly encourage code deposition in a community repository (e.g. GitHub). See the Nature Portfolio guidelines for submitting code & software for further information.

Data

Policy information about availability of data

All manuscripts must include a data availability statement. This statement should provide the following information, where applicable:

- Accession codes, unique identifiers, or web links for publicly available datasets
- A description of any restrictions on data availability
- For clinical datasets or third party data, please ensure that the statement adheres to our policy

Raw sequencing data have been deposited in the NCBI Sequence Read Archive (SRA) under accession PRJNA1315251. This can be accssed via the following link: https://dataview.ncbi.nlm.nih.gov/object/PRJNA1315251?reviewer=9r3eud2qsgof0rrld2rq0guvdo. All other data associated with this study are present in the paper or the Supplementary Materials. The source data for Table 1, Figures 3a, 4, S2a, 3, and S4 is in Supplementary Data 'Source Data' file.

Research involving human participants, their data, or biological material

Policy information about studies with human participants or human data. See also policy information about sex, gender (identity/presentation), and sexual orientation and race, ethnicity and racism.

Reporting on sex and gender	The cohort included 15 samples of liquid biopsy in 10 patients (female 7, male 3) with extracranial AVMs who had a mean age of 12.9 years (SD 4.76, range 7.3–22.2 years) at the time of biopsy.
Reporting on race, ethnicity, or other socially relevant groupings	No reporting undertaken on this
Population characteristics	The cohort included 15 samples of liquid biopsy in 10 patients (female 7, male 3) with extracranial AVMs who had a mean age of 12.9 years (SD 4.76, range 7.3–22.2 years) at the time of biopsy. The anatomical locations of AVM involvement included the face (n=4), fingers (2), pelvis (1), lower limb (1), and spinal cord with additional involvement of the overlying metameric skin and paraspinal tissues (2: spinal arteriovenous metameric syndrome – SAMS; formerly termed Cobb syndrome). All 10 patients had previously undergone endovascular treatment(s) without successful angiographic or clinical cure and had persistent symptoms or disability relating to their condition.
Recruitment	• Study design and enrolment/consent<br>This was a prospective single-arm cohort study of paediatric and young adult patients (all of age <18 years at time of initial diagnosis and treatment) with arterio-venous malformations (AVMs) causing disabling symptoms despite endovascular +/- surgical therapy. The primary aim was to assess for the presence of somatic mosaic variants involving the RAS-MEK and PI3K-mTOR pathways in paediatric patients with AVMs. The secondary aim was to further confirm the feasibility of transvenous liquid biopsy of blood from the efferent draining vein of AVMs as a method of assessing for somatic mosaic variants via cfDNA extraction.
Ethics oversight	• Ethics<br>Ethics approval for this study was granted by the Sydney Children's Hospital Network Human Research Ethics Committee (2021/ETH12371). Informed written consent to participate in the study was obtained from the patient or their primary caregiver for all participants. All study procedures were undertaken according to principles of the Declaration of Helsinki.

Note that full information on the approval of the study protocol must also be provided in the manuscript.

Field-specific reporting

Please select the one below that is the best fit for your research. If you are not sure, read the appropriate sections before making your selection.

☒ Life sciences ☐ Behavioural & social sciences ☐ Ecological, evolutionary & environmental sciences

For a reference copy of the document with all sections, see nature.com/documents/nr-reporting-summary-flat.pdf

Life sciences study design

All studies must disclose on these points even when the disclosure is negative.

Sample size	The cohort included 15 samples of liquid biopsy in 10 patients (female 7, male 3) with extracranial AVMs who had a mean age of 12.9 years (SD 4.76, range 7.3–22.2 years) at the time of biopsy. The presence of 15 samples was accounted for by the collection of two separate draining vein samples each from two patients at separate treatment session, three patients having additional peripheral blood testing, and one patient having only a peripheral blood sample tested.
Data exclusions	None
Replication	• Orthogonal confirmation<br>i) ddPCR – NM_004985(KRAS):c.35G>A (p.G12D) [KRAS p.G12D] was analysed using PrimePCR variant detection assay dHsaMDV2510596 (KRAS:c.35G>A, p.G12D, 6-FAM, KRAS WT reference gene, HEX; Bio-Rad Laboratories) as described in Kahana-Edwin et al., 202144. Controls included non-template control (NTC) which contained purified water instead of cfDNA, and the artificial cfDNA reference standards described above (Horizon Discovery). The ddPCR reaction mixture was used for droplet generation, and amplification was carried out in a C1000 Touch Thermal Cycler (Bio-Rad Laboratories) under the following conditions: 95 °C for 10 min, 40 cycles of 94 °C for 30 s, 55 °C for 1 min; then 98 °C for 10 min. ddPCR was performed using the QX200 ddPCR system according to manufacturer's instructions (Bio-Rad Laboratories). QuantaSoft™ Analysis Pro v1.0 software (Bio-Rad Laboratories) used for data analysis. Target and reference/s copies were within the dynamic range of the instrument to ensure accurate detection level.<br>ii) FFPE genomic DNA underwent MPS library preparation and sequencing as described above.
Randomization	Not undertaken
Blinding	Not undertaken

Reporting for specific materials, systems and methods

We require information from authors about some types of materials, experimental systems and methods used in many studies. Here, indicate whether each material, system or method listed is relevant to your study. If you are not sure if a list item applies to your research, read the appropriate section before selecting a response.

Materials & experimental systems

n/a	Involved in the study
☒	☐ Antibodies
☒	☐ Eukaryotic cell lines
☒	☐ Palaeontology and archaeology
☒	☐ Animals and other organisms
☐	☒ Clinical data
☒	☐ Dual use research of concern
☒	☐ Plants

Methods

n/a	Involved in the study
☒	☐ ChIP-seq
☒	☐ Flow cytometry
☒	☐ MRI-based neuroimaging

Clinical data

Policy information about clinical studies

All manuscripts should comply with the ICMJE guidelines for publication of clinical research and a completed CONSORT checklist must be included with all submissions.

Clinical trial registration	Not a clinical trial
Study protocol	Not a clinical trial
Data collection	This was a prospective single-arm cohort study of paediatric and young adult patients (all of age <18 years at time of initial diagnosis and treatment) with arterio-venous malformations (AVMs) causing disabling symptoms despite endovascular +/- surgical therapy. The primary aim was to assess for the presence of somatic mosaic variants involving the RAS-MEK and PI3K-mTOR pathways in paediatric patients with AVMs. The secondary aim was to further confirm the feasibility of transvenous liquid biopsy of blood from the efferent draining vein of AVMs as a method of assessing for somatic mosaic variants via cfDNA extraction.
Outcomes	This was a prospective single-arm cohort study of paediatric and young adult patients (all of age <18 years at time of initial diagnosis and treatment) with arterio-venous malformations (AVMs) causing disabling symptoms despite endovascular +/- surgical therapy. The primary aim was to assess for the presence of somatic mosaic variants involving the RAS-MEK and PI3K-mTOR pathways in paediatric patients with AVMs. The secondary aim was to further confirm the feasibility of transvenous liquid biopsy of blood from the efferent draining vein of AVMs as a method of assessing for somatic mosaic variants via cfDNA extraction.

Plants

Seed stocks	*Report on the source of all seed stocks or other plant material used. If applicable, state the seed stock centre and catalogue number. If plant specimens were collected from the field, describe the collection location, date and sampling procedures.*
Novel plant genotypes	*Describe the methods by which all novel plant genotypes were produced. This includes those generated by transgenic approaches, gene editing, chemical/radiation-based mutagenesis and hybridization. For transgenic lines, describe the transformation method, the number of independent lines analyzed and the generation upon which experiments were performed. For gene-edited lines, describe the editor used, the endogenous sequence targeted for editing, the targeting guide RNA sequence (if applicable) and how the editor was applied.*
Authentication	*Describe any authentication procedures for each seed stock used or novel genotype generated. Describe any experiments used to assess the effect of a mutation and, where applicable, how potential secondary effects (e.g. second site T-DNA insertions, mosiacism, off-target gene editing) were examined.*
